# Obesity, skin disorders, and the microbiota: Unraveling a complex web

**DOI:** 10.71150/jm.2508007

**Published:** 2026-01-31

**Authors:** Yu Ri Woo, Hei Sung Kim

**Affiliations:** Department of Dermatology, Incheon St. Mary’s Hospital, College of Medicine, The Catholic University of Korea, Seoul 06649, Republic of Korea

**Keywords:** obesity, hidradenitis suppurativa, psoriasis, gut–skin axis, microbiota, dysbiosis, Th17, IL-22

## Abstract

Obesity is increasingly recognized as a systemic pro-inflammatory condition that influences not only metabolic and cardiovascular health but also the development and exacerbation of cutaneous inflammatory diseases. This review examines the interplay between obesity, microbial dysbiosis, and two archetypal inflammatory skin disorders—hidradenitis suppurativa (HS) and psoriasis. We highlight how obesity-induced changes in immune signaling, gut permeability, and microbiota composition—both in the gut and the skin—contribute to cutaneous inflammation. Special emphasis is placed on shared pathways such as the Th17/IL-23 and IL-22 signaling axes, adipokine imbalance, and microbial metabolites like short-chain fatty acids and lipopolysaccharides. The review critically evaluates the current literature, distinguishing preclinical insights from clinical evidence, and underscores the potential of microbiota-targeted therapies and metabolic interventions as adjunctive treatment strategies. By integrating metabolic, immunologic, and microbiome data, we synthesize emerging evidence to better understand the gut–skin–obesity interplay and guide future therapeutic innovations.

## Introduction

Obesity, both a chronic inflammatory condition and metabolic disorder, has become a major global health concern with rising prevalence. Obesity is defined by the World Health Organization as a body mass index (BMI) of 30 kg/m^2^ or more, and is linked to increased risks of cardiovascular disease, insulin resistance, and systemic inflammation. Beyond these metabolic outcomes, obesity exerts profound effects on the skin—both structurally and immunologically—manifesting in increased severity and prevalence of various dermatologic conditions. Among these, hidradenitis suppurativa (HS) and psoriasis stand out as chronic inflammatory dermatoses most consistently linked to obesity, both epidemiologically and mechanistically (Hirt et al., 2019).

Recent advances have highlighted the role of the human microbiota—particularly that of the gut and skin—as a key mediator in the bidirectional relationship between obesity and these inflammatory skin diseases. Microbial dysbiosis may serve as both a marker and a modulator of disease activity, with alterations in microbial metabolites, such as short-chain fatty acids (SCFAs) and lipopolysaccharide (LPS), contributing to systemic inflammation and immune dysregulation (Asadi et al., 2022). Simultaneously, the gut-skin axis has emerged as a compelling model to explain how microbial and metabolic disturbances in obesity can impact cutaneous immunity (Aron-Wisnewsky et al., 2021).

Among the most critical immunologic links is the activation of the Th17/interleukin (IL)-23 and IL-22 pathways, which promote keratinocyte proliferation, barrier disruption, and inflammatory cytokine release (Hirt et al., 2019). These axes serve as common mechanistic threads in the pathogenesis of HS and psoriasis, both of which are characterized by neutrophilic inflammation and perturbed barrier function.

While prior integrative reviews have primarily emphasized on skin physiology (Darlenski et al., 2022; Yosipovitch et al., 2007), this article differs by systematically synthesizing recent skin and gut microbiome evidence, including metagenomic and functional studies, contrasting obesity-driven versus disease-specific microbial changes, and discussing therapeutic strategies that target the microbiota–metabolism–skin axis. In doing so, this review positions the microbiome as a key link in the gut-skin-obesity triad and evaluates its translational potential for novel interventions.

## Changes in Skin Physiology in Obese Patients

In individuals with obesity, the skin undergoes a variety of functional and structural changes resulting from both local and systemic effects of excessive adiposity. These changes are not superficial manifestations but reflect the pathophysiological consequences of metabolic inflammation, immune dysregulation, and microbiota alterations. Understanding these changes is essential for clarifying how obesity contributes to skin disease onset, severity, and chronicity.

### Impaired skin barrier function

Obesity-related shifts in epidermal physiology affect skin barrier integrity and hydration, though study findings remain mixed. Transepidermal water loss (TEWL) has been positively associated with BMI in recent studies, and Mendelian randomization data suggest a causal link (Yew et al., 2023). This impairment may be linked to obesity-induced low-grade inflammation, altered lipid synthesis, and cytokine-driven disruption of tight junction proteins—especially via IL-6, tumor necrosis factor (TNF)-α, and IL-22, which are overexpressed in obesity and known to affect keratinocyte differentiation (Hirt et al., 2019).

### Sebaceous and apocrine/eccrine gland activity

Obesity alters sebaceous, eccrine, and apocrine gland function through hormonal and adipokine imbalances. Elevated insulin-like growth factors and androgens stimulate sebaceous proliferation and sebum production, while increased leptin and reduced adiponectin promote sebocyte inflammation (Abulnaja, 2009; Deplewski and Rosenfield, 1999; Izquierdo et al., 2019; Jung et al., 2017). Eccrine activity is heightened, causing excessive sweating in skin folds and favouring microbial shifts (Adams et al., 2015). Apocrine secretions, metabolized by skin microbiota, contribute to body odour, with axillary osmidrosis more common in individuals with high BMI (Dong et al., 2021).

### Wound healing and collagen remodeling

Obesity is associated with impaired wound healing and altered collagen architecture. Insufficient collagen synthesis relative to skin expansion leads to reduced tensile strength and disorganized fibers, as shown in both human and animal studies (do Nascimento and Monte-Alto-Costa, 2011; Enser and Avery, 1984; Matsumoto et al., 2014). Chronic low-grade inflammation in adipose tissue alters fibroblast activity and increases the expression of matrix metalloproteinases, contributing to defective collagen deposition and tissue remodeling (Pierpont et al., 2014). Concurrently, vascular insufficiency and localized hypoxia further delay re-epithelialization and collagen maturation, while skin microbial dysbiosis and reduced innate immunity heighten infection risk (Larouche et al., 2018; Pierpont et al., 2014). Collectively, these factors create an adverse dermal microenvironment that hinders effective tissue repair.

### Vascular and lymphatic alterations

Obesity impairs cutaneous vascular and lymphatic function. Microvascular reactivity is reduced, driven by elevated free fatty acids (FFAs), TNF-α, and decreased adiponectin, which compromise endothelial integrity (de Jongh et al., 2004; Kern et al., 2003). Venous hypertension from intra-abdominal pressure and valve incompetence increases vascular stress, contributing to stasis dermatitis, varicose veins, and atrophie blanche (Danielsson et al., 2002; Sugerman, 2001). Excess adiposity also disrupts lymphatic drainage, causing edema, hypoxia, and cytokine accumulation (Kataru et al., 2020). Mechanistically, obesity reduces lymphatic vessel contractility, increases endothelial permeability, and alters lymphatic endothelial cell (LEC) gene expression, thereby sustaining inflammation and tissue remodeling, which may aggravate conditions such as HS and chronic venous insufficiency (Kataru et al., 2020).

### Skin microbiota in obesity

Healthy human skin harbors a diverse array of microbial communities, with composition varying by anatomical site, sebaceous activity, and environmental exposure. Dominant bacterial genera in healthy skin includes *Cutibacterium*, *Staphylococcus*, *Corynebacterium*, and fungi such as *Malassezia*. In healthy skin, the volar forearm skin microbiome is predominantly composed of *Cutibacterium acnes*, *Staphylococcus epidermidis*, and various *Streptococcus* species (Shi et al., 2016), and post-pubertal increases in sebum production selectively support colonization by lipophilic microbes like *Cutibacterium* and *Corynebacterium*.

Despite the known impact of obesity on gut microbiota, relatively few studies have explored its effect on skin microbiome in obesity (Brandwein et al., 2019; Ma et al., 2024; Rood et al., 2018; Vongsa et al., 2019; Walker et al., 2020). In a 2019 exploratory study, 40 female participants were stratified by BMI to compare microbial composition in exposed (abdominal) versus unexposed (vulvar) skin (Vongsa et al., 2019). High BMI was associated with elevated pH and increased colonization by *Finegoldia* and *Corynebacterium* in the vulvar region, while abdominal skin showed minimal BMI-related differences (Vongsa et al., 2019). This suggests that obesity may more profoundly affect microbiota in occluded or moist anatomical regions.

Larger cohort studies further support obesity-associated shifts in cutaneous microbial composition. An analysis of 822 skin samples from the American Gut Project found that individuals with lower BMI harbored greater microbial diversity across various skin sites, with underweight individuals showing broader microbial richness than those with obesity (Brandwein et al., 2019). The study also reported a positive correlation between BMI and the abundance of *Corynebacterium*, reinforcing the potential role of adiposity in promoting the colonization of specific microbial taxa (Brandwein et al., 2019). A recent cohort study in China, examined the facial skin microbiome and physiological parameters in 198 healthy women aged 18 to 35 years (Ma et al., 2024). This study found that higher BMI was associated with impaired skin barrier function, indicated by increased TEWL and decreased skin surface pH (Ma et al., 2024). Notably, facial skin bacterial and fungal diversity was increased in the overweight group, with higher abundances of *Streptococcus*, *Corynebacterium*, *Malassezia*, and *Candida* compared to individuals with normal BMI (Ma et al., 2024). There were also significant negative correlations between skin pH and relative abundances of *Malassezia* and *Candida*, suggesting complex interactions between skin physiology and microbial communities (Ma et al., 2024). Unlike Brandwein et al. (2019)’s study, which surveyed multiple skin sites mainly in a U.S. population, the Shanghai study focused on facial skin in an East Asian cohort. The findings underscore how anatomical site, ethnicity, and geographic context can shape obesity-associated changes in the skin microbiota.

Together, these findings indicate that obesity alters cutaneous microbial communities by modifying the chemical and physical microenvironment of the skin. Changes in skin surface pH, hydration, sweat composition, and occlusion may collectively shape microbial diversity and promote the overgrowth of *Corynebacterium* and other opportunistic taxa. These obesity-related microbial and physiological shifts may predispose the skin to inflammation, barrier disruption, and dysbiosis, potentially amplifying the severity of obesity-associated dermatoses (Table 1).

## Obesity and Inflammatory Skin Disorders

**Fig. 1.**
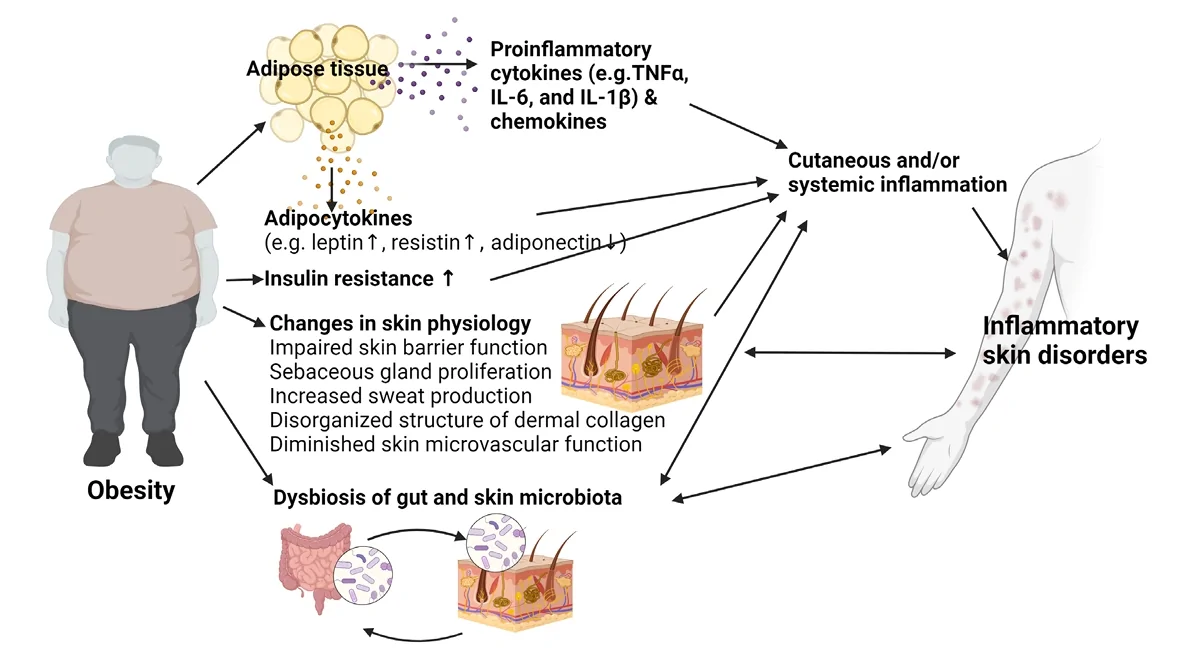
Schematic illustration depicting the bidirectional interactions between obesity and inflammatory skin diseases, highlighting shared immunologic, metabolic, and microbial pathways.

Obesity is increasingly recognized as a systemic inflammatory and metabolic condition that extends its influence far beyond traditional cardiometabolic comorbidities. Accumulating evidence demonstrates that excess adiposity has profound effects on cutaneous immunity and disease expression, contributing to both the onset and exacerbation of chronic inflammatory skin disorders (Fig. 1). Among these, HS and psoriasis are the conditions most consistently linked to obesity, with both epidemiologic and mechanistic studies supporting a strong bidirectional relationship.

Epidemiologic data consistently demonstrate higher prevalence and increased severity of both HS and psoriasis in individuals with obesity (Carrascosa et al., 2014; Kromann et al., 2014). Obesity also reduces responsiveness to biologics and conventional systemic therapies, likely due to altered pharmacokinetics, intensified baseline inflammation, and persistent metabolic dysfunction. This bidirectional interplay between skin disease and obesity—where chronic inflammation contributes to sedentary behavior, weight gain, and worsening metabolic health, creates a reinforcing cycle that complicates long-term management.

Although HS and psoriasis present distinct clinical phenotypes—one characterized by follicular occlusion followed by rupture and secondary immune activation (Krajewski et al., 2023), the other by epidermal hyperproliferation and plaque formation (Carrascosa et al., 2014)—both diseases are strongly shaped by obesity-related immunometabolic disturbances. Excess adipose tissue actively produces proinflammatory cytokines (e.g., TNF-α, IL-6, IL-1β) and chemokines. At the same time, obesity induces characteristic alterations in adipokine secretion—characterized by elevated leptin and resistin and reduced adiponectin—promotes Th1/Th17 polarization, NF-κB activation, and metabolic inflammation that drives insulin resistance (González-López et al., 2020; Kyriakou et al., 2018). These systemic alterations converge on inflammatory pathways implicated in HS and psoriasis, particularly the Th17/IL-23 axis, which drives keratinocyte proliferation, neutrophil recruitment, and barrier disruption (Fletcher et al., 2020; van Straalen et al., 2022).

Leptin promotes Th1/Th17 differentiation and correlates with disease severity; resistin amplifies NF-κB–mediated inflammation; and reduced adiponectin decreases IL-10 and weakens counter-regulation of TNF-α and IL-6 (González-López et al., 2020; Kyriakou et al., 2018; Malara et al., 2018; Seth et al., 2020). These obesity-associated adipokine shifts reinforce systemic inflammation that, in turn, amplifies cutaneous immune activation. Elevated levels of IL-1β, IL-6, IL-17, and TNF-α contribute to follicular rupture and sinus tract formation in HS (Krajewski et al., 2023), and drive keratinocyte proliferation and plaque development in psoriasis (Carrascosa et al., 2014).

Obesity also alters fundamental aspects of skin physiology, further linking excess weight to disease severity. Mechanical stress in intertriginous regions, exacerbated by central adiposity, follicular occlusion, and microinjury in HS (Mintoff et al., 2023), while impaired barrier function and altered hydration states associated with obesity can intensify psoriatic inflammation.

Microbiome-level changes further reinforce these mechanisms. Obesity-associated gut dysbiosis, characterized by increased intestinal permeability and metabolic endotoxemia, leads to enhanced LPS–TLR4 signaling and Th17-skewed immunity (Cani et al., 2007; Wang et al., 2012). Concurrently, skin microbiome alterations—including increased *Corynebacterium*, *Finegoldia*, and *Staphylococcus* in occluded sites—mirror patterns observed in HS and psoriasis (Chang et al., 2018; Vongsa et al., 2019), suggesting shared microbial pathways linking systemic metabolism to local cutaneous inflammation.

Obesity-driven immunometabolic disturbances shape the inflammatory milieu of both HS and psoriasis, but these effects are further compounded by disease-specific and shared alterations in the cutaneous microbiome. Because the skin’s microbial communities interact intimately with the barrier, sebaceous and sweat gland function, and local immune signaling—all of which are profoundly modulated by obesity—microbiome dysregulation has emerged as a critical mechanistic bridge linking systemic metabolic dysfunction with cutaneous inflammation. The following section reviews how obesity intersects with disease-specific patterns of skin microbiota dysbiosis in HS and psoriasis and how these microbial changes may contribute to disease chronicity and severity.

## Altered Skin Microbiota in Inflammatory Skin Disorders

Obesity-driven immunometabolic disturbances shape the inflammatory milieu of both HS and psoriasis, but these effects are further compounded by disease-specific and shared alterations in the cutaneous microbiome. Because the skin’s microbial communities interact intimately with the barrier, sebaceous and sweat gland function, and local immune signaling—all of which are profoundly modulated by obesity—microbiome dysregulation has emerged as a critical mechanistic bridge linking systemic metabolic dysfunction with cutaneous inflammation. The following section reviews how obesity intersects with disease-specific patterns of skin microbiota dysbiosis in HS and psoriasis and how these microbial changes may contribute to disease chronicity and severity.

Growing evidence indicates that dysbiosis of the skin microbiota is a shared feature of inflammatory skin diseases, including HS and psoriasis. Although these disorders present distinct clinical and immunologic profiles, both exhibits reduced microbial diversity, expansion of opportunistic or inflammation-associated taxa, and depletion of protective commensals (Guet-Revillet et al., 2017; Haskin et al., 2016; Naik et al., 2020). These alterations interact with obesity-related changes in skin physiology—such as increased humidity in skin folds, altered sebum composition, and barrier impairment—to amplify local inflammation and disease chronicity.

Across both HS and psoriasis, multiple studies report an increased abundance of *Corynebacterium*, *Staphylococcus*, and various facultative or obligate anaerobes, along with a consistent reduction in *Cutibacterium*, a dominant commensal associated with lipid homeostasis and barrier integrity (Chang et al., 2018; Schneider et al., 2020). The loss of *Cutibacterium* may weaken colonization resistance and enhance antigenic exposure to the immune system, contributing to heightened local inflammation.

Obesity appears to accentuate these dysbiotic patterns. Obese HS patients, for example, are significantly more likely to exhibit *Firmicutes*-dominant skin flora compared with non-obese HS patients (Haskin et al., 2016). Similar BMI-associated increases in *Corynebacterium* and *Staphylococcus* have been observed in occluded or high-sebum regions in other cohorts, suggesting shared microenvironmental effects of obesity across inflammatory dermatoses.

HS shows a particularly prominent enrichment of anaerobic, proteolytic, and biofilm-forming bacteria in intertriginous regions (Ring et al., 2019). Studies consistently report increased representation of *Porphyromonas*, *Prevotella*, *Peptoniphilus*, *Anaerococcus*, *Finegoldia magna*, and *Fusobacterium* in HS lesions (Guet-Revillet et al., 2017; McCarthy et al., 2022; Ring et al., 2017). Importantly, taxa such as *Fusobacterium* and *Parvimonas* correlate with greater disease severity, especially in Hurley stage III disease. Notably, lesional and non-lesional HS skin often share similar dysbiotic profiles (Schneider et al., 2020), reflecting a “field effect” driven by chronic inflammation, occlusion, and persistent microenvironmental dysfunction. This pattern is characterized by enrichment of opportunistic anaerobes—such as *Porphyromonas*, *Anaerococcus*, and *Peptoniphilus*—together with a marked depletion of *Cutibacterium* (Schneider et al., 2020).

In psoriasis, dysbiosis is typically characterized by altered proportions of aerobic or aerotolerant genera. Lesional plaques often display increased levels of *Streptococcus*, *Staphylococcus*, and *Corynebacterium* alongside reduced *Cutibacterium* (Chang et al., 2018). Emerging multi-omics data highlight *Corynebacterium simulans* as a taxon linked to antiviral/interferon pathways and greater disease severity. Therapeutic modulation of the microbiome has also been observed: IL-17A inhibitor treatment partially restores functional microbial pathways, with improvements in methionine and tryptophan metabolism aligning toward healthy skin profiles (Lv et al., 2025).

**Fig. 2.**
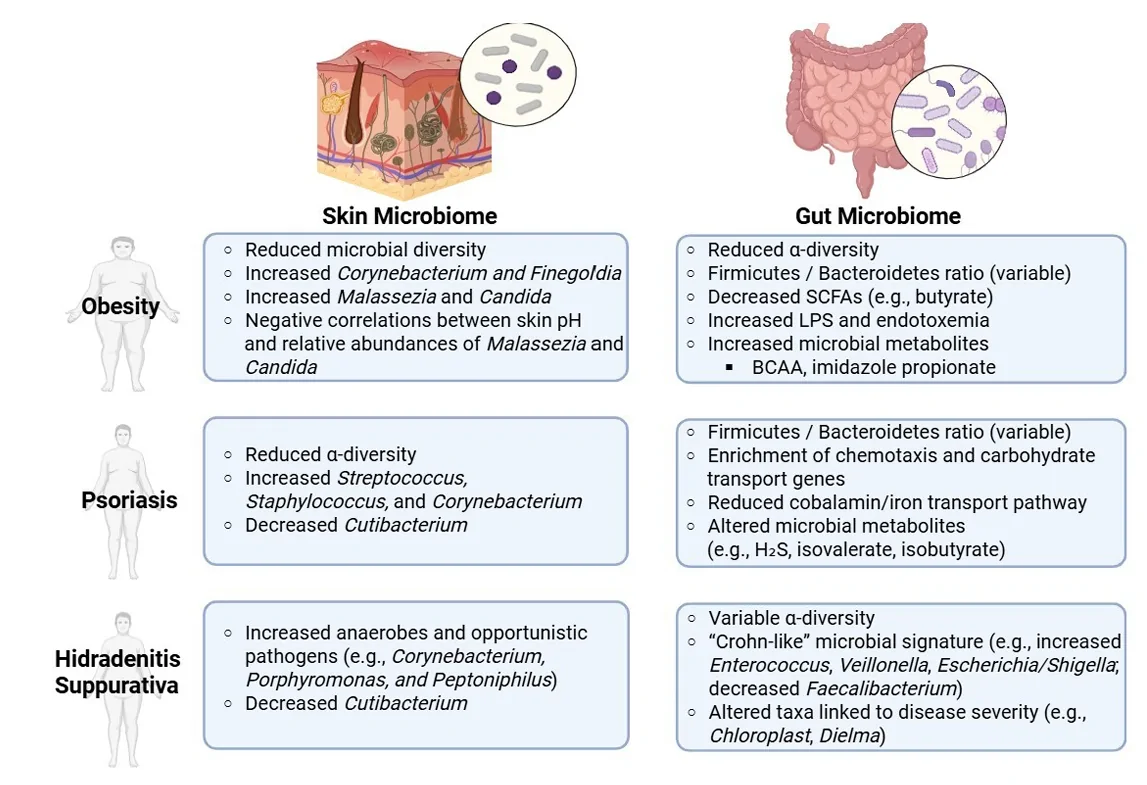
Alterations in the skin and gut microbiome across obesity, psoriasis, and hidradenitis suppurativa (HS).

Collectively, these findings show that while HS demonstrates an anaerobe-dominant, biofilm-rich dysbiosis, and psoriasis a *Streptococcus*/*Staphylococcus*/*Corynebacterium*-enriched profile, both share key unifying features (Fig. 2). These overlapping microbial perturbations likely contribute to impaired barrier function, enhanced antigen presentation, and persistent immune activation, helping to explain the chronic, relapsing nature of both HS and psoriasis—particularly in individuals with obesity.

## Altered Gut Microbiota in Obesity

Obesity-associated gut dysbiosis is not defined by a uniform phylum-level signature—*Firmicutes*/*Bacteroidetes* shifts are heterogeneous across cohorts and methods—whereas small but recurrent reductions in α-diversity and, more robustly, functional perturbations (e.g., impaired SCFA production, lower butyrate-producing capacity, increased endotoxaemia, bile-acid/indole pathway changes) are observed more consistently (Cheng et al., 2022; Coppola et al., 2021; Duncan et al., 2008; Koliada et al., 2017; Ley et al., 2006; Sze and Schloss, 2016). Causality is supported by gnotobiotic transfers: obese-donor communities increase adiposity in germ-free mice and transmit an “obese” phenotype in twin-discordant models unless outcompeted by lean taxa (Ridaura et al., 2013; Turnbaugh et al., 2006).

High-fat feeding increases gut permeability and lipopolysaccharide (LPS), driving “metabolic endotoxaemia”; blocking LPS–CD14/TLR4 protects against weight gain/insulin resistance (Cani et al., 2007). High-dose LPS can drive Th17 differentiation through dendritic-cell–dependent signaling, suggesting that LPS elevations in obesity may similarly enhance Th17-mediated systemic and cutaneous inflammation (Wang et al., 2012).

In addition, butyrate induces colonic FOXP3⁺ Tregs/IL-10 via histone deacetylase (HDAC) inhibition, while propionate/succinate trigger intestinal gluconeogenesis and a portal–vagal circuit improving glycaemia and energy balance—pathways that can temper Th17/IL-23 tone (De Vadder et al., 2014; Furusawa et al., 2013; Yoshida et al., 2019).

Gut microbes transform bile acids, which can signal through FXR and TGR5 pathways. This stimulates intestinal L-cells to release glucagon-like peptide (GLP)-1, enhancing satiety and glucose control and thereby supporting resistance to obesity. Antibiotic disruption of the microbiota abolishes these benefits (Pathak et al., 2018). Bariatric surgery remodels BA–microbiota networks, and post-operative communities transfer leanness to mice (Liou et al., 2013).

Bacterial indoles activate aryh hydrocarbon receptor (AhR), boosting IL-22 and intestinal barrier repair, a mechanism that may protect against obesity-associated low-grade inflammation (Zelante et al., 2013). In contrast, gut dysbiosis in obesity can enrich pathways for branched-chain amino acid (BCAA) biosynthesis (notably linked to *Prevotella copri*) and increase levels of imidazole propionate, a microbial metabolite that impairs insulin signalling and reduces responsiveness to metformin, thereby exacerbating obesity-related insulin resistance and metabolic dysfunction (Koh et al., 2018; Natividad et al., 2018; Pedersen et al., 2016; Zelante et al., 2013).

## Altered Gut Microbiota in Inflammatory Skin Disorders

Growing evidence suggests that gut microbial dysbiosis contributes to the systemic immune activation observed in both psoriasis and HS. Although findings vary across studies due to differences in geography, sequencing approaches, and the strong confounding impact of obesity, several reproducible taxonomic and functional signatures have emerged across these conditions. Overall, psoriasis and HS both displays altered microbial composition, disrupted metabolic pathways, and enrichment of proinflammatory microbial metabolites, supporting the existence of an intestinal inflammatory axis that may influence cutaneous disease expression.

Many cohorts demonstrate an increased *Firmicutes*-to-*Bacteroidetes* ratio (Chen et al., 2018; Dei-Cas et al., 2020; Shapiro et al., 2019), although some studies report the opposite shift (Huang et al., 2019; Wen et al., 2023), underscoring the influence of population- and diet-related variability. Functional analyses consistently indicate over-representation of pathways related to chemotaxis and carbohydrate transport (Chen et al., 2018), lipopolysaccharide biosynthesis, WNT signaling, apoptosis, bacterial secretion systems, and phosphotransferase systems (Chen et al., 2018; Xiao et al., 2021). Psoriatic fecal metabolomic profiles frequently show elevated levels of hydrogen sulfide, isovalerate, isobutyrate, hyaluronan, and hemicellulose (Xiao et al., 2021), suggesting heightened microbial metabolic activity that may promote epithelial dysfunction and amplify immune activation. Importantly, when analyses are adjusted for BMI, microbial alterations become more distinct, indicating that obesity may obscure psoriasis-specific dysbiosis (Chen et al., 2018). Therefore, longitudinal and multi-omics studies in lean psoriasis cohorts will be essential to disentangle adiposity-related effects from disease-specific microbial signatures.

HS also exhibits significant gut microbial dysregulation, although the pattern differs somewhat from psoriasis. Several studies describe reduced alpha diversity in HS (Kam et al., 2021; Öğüt et al., 2022), while others report comparable diversity but distinct compositional shifts (Lelonek et al., 2025). Specifically, HS was linked to reduced abundance of several commensal taxa (e.g., *Collinsella*, *Clostridia*, *Lachnospiraceae*) and enrichment of potentially pathogenic or opportunistic genera (e.g., *Enterorhabdus*, *Holdemanella*, *Comamonas*, *Enterobacter*) (Lelonek et al., 2025). Disease severity further influenced microbial composition, with higher odds of occurrence reported for certain genera such as *Chloroplast*, *Dielma*, *Eisenbergiella*, and *Paludicola* (Lelonek et al., 2025). Approximately 40% of HS patients possess a “Crohn-like” gut microbial signature—a configuration marked by enrichment of pathogenic genera such as *Enterococcus*, *Veillonella*, *Escherichia*/*Shigella*, and a pronounced loss of beneficial taxa like *Faecalibacterium* (Cronin et al., 2023). This microbial pattern is associated with decreased levels of the anti-inflammatory mediator growth-arrest specific 6 (GAS6) and elevated systemic inflammatory cytokines, particularly interleukin-12 (IL-12) (Cronin et al., 2023), suggesting potential overlap between HS and gastrointestinal inflammatory disorders.

Additional findings further support the concept of microbiome-driven immune dysregulation in HS. The absence of *Bifidobacterium adolescentis* (*B. adolescentis*) in adults but its presence inf all pediatric HS patients, suggests disrupted age-related maturation of the gut microbiome (Collard et al., 2025). In addition, causal inference analysis suggested that specific gut taxa may differentially influence HS risk: the *Clostridium innocuum* group and *Lachnospira* were associated with potential risk-promoting (anti-protective) effects, whereas *Family XI* and *Porphyromonadaceae* appeared to confer protective influences (Liu et al., 2024). Collectively, these observations suggest that HS-associated gut dysbiosis is defined less by uniform reductions in diversity and more by distinct taxonomic and functional disruptions that track with disease severity and systemic inflammation.

Taken together, the gut microbiome in psoriasis and HS displays both shared and disease-specific abnormalities. Both conditions exhibit functional dysbiosis characterized by increased LPS-related pathways, altered SCFA-related metabolism, enrichment of inflammatory microbial metabolites, and depletion of beneficial commensals (Cani et al., 2007; Cronin et al., 2023; Xiao et al., 2021). Psoriasis is more frequently associated with perturbations in *Firmicutes* and shifts in carbohydrate- and LPS-related metabolic pathways (Chen et al., 2018; Xiao et al., 2021), while HS shows stronger links to Crohn-like signatures, loss of *Faecalibacterium*, and enrichment of proinflammatory pathobionts (Cronin et al., 2023; Lelonek et al., 2025). These convergent microbial disturbances may promote systemic inflammation, enhance Th17/IL-23 signaling (Wang et al., 2012), and contribute to the chronic, relapsing nature of both skin diseases—particularly in individuals with obesity, where metabolic endotoxemia and increased intestinal permeability further amplify gut–skin immune cross-talk (Cani et al., 2007).

## Therapeutic Approaches and Future Directions

### Lifestyle modifications and weight management

Given the strong correlation between obesity and various dermatologic conditions, weight management forms the cornerstone of therapeutic interventions. Numerous studies suggest that weight reduction can significantly improve disease severity in both HS and psoriasis. A comprehensive review reported that weight loss interventions are associated with symptom improvement in HS, especially in friction-prone areas (Boer, 2016; Sivanand et al., 2020). In psoriasis, a meta-analysis demonstrated that non-pharmacologic weight reduction strategies lead to significant improvement in disease severity among overweight and obese individuals (Upala and Sanguankeo, 2015).

Because dietary composition is one of the strongest determinants of gut microbial structure, dietary modifications such as low glycemic-load diets and adherence to Mediterranean dietary patterns have shown benefit in reducing systemic inflammation and improving skin conditions associated with metabolic syndrome. These lifestyle interventions likely exert their effects not only through metabolic pathways but also through reshaping gut microbial communities.

### Microbiota-targeted therapies

With growing recognition of the gut–skin axis, microbiota-targeted interventions—including prebiotics, probiotics, synbiotics, and postbiotics—are gaining attention as potential adjunctive therapies for obesity-associated skin diseases. These interventions aim to restore microbial balance in both the gut and skin, thereby mitigating systemic inflammation and improving cutaneous outcomes.

In psoriasis, a randomized controlled trial showed that a 12-week oral probiotic formulation containing *Bifidobacterium longum* CECT 7347, *B. lactis* CECT 8145, and *Lactobacillus rhamnosus* CECT 861 resulted in up to a 75% reduction in PASI scores (Navarro-López et al., 2019). Although clinical evidence specifically targeting obesity-associated skin diseases is limited, shared features of dysbiosis across obesity, psoriasis, and HS—such as reduced microbial diversity, a lower abundance of *Bacteroidetes*, and a relative increase in *Firmicutes*—provide strong biological plausibility.

Microbiota-directed interventions may benefit both obesity and skin inflammation through convergent immunometabolic pathways. Probiotics, prebiotics, and dietary fiber supplementation increase the production of short-chain fatty acids (SCFAs), enhance intestinal barrier integrity, and reduce metabolic endotoxemia (Abulnaja, 2009; Li et al., 2024; Mishra et al., 2023; Zhao et al., 2018), thereby suppressing systemic low-grade inflammation that drives metabolic dysfunction and Th17-skewed skin inflammation. Restoration of microbial diversity also modulates IL-23/IL-17 signaling and improves adipose–immune crosstalk, offering a unified mechanism through which microbial modulation can influence both metabolic and dermatologic outcomes.

Bidirectional interactions between the skin barrier and gut microbiome While it is well established that gut dysbiosis can influence skin inflammation, emerging data suggest that skin barrier disruption can also affect gut microbial ecology. In a landmark study, Dokoshi et al. (2024) demonstrated that mechanical skin injury or epidermal overexpression of hyaluronidase in mice led to significant alterations in the gut microbiota, including reduced diversity, loss of beneficial species, and increased susceptibility to DSS-induced colitis. Mechanistically, skin damage released dermal hyaluronan fragments, which acted as damage-associated molecular patterns (DAMPs), inducing intestinal expression of Reg3 and Muc2, and altering colon microbial composition—even in germ-free mice (Dokoshi et al., 2024). These findings underscore the complex and reciprocal nature of the microbiota–metabolism–skin network, in which interventions targeting one barrier tissue may have therapeutic consequences for another.

### Pharmacological interventions

Pharmacologic treatments that target metabolic dysfunction not only improve weight and glycemic control but may also alleviate chronic inflammatory skin conditions via immunometabolic and potentially microbiome-mediated mechanisms. Table 2 highlights key studies evaluating the therapeutic effects of various metabolic-targeting interventions on obesity-associated dermatoses.

A meta-analysis confirmed the efficacy of pioglitazone, an oral antidiabetic agent, in improving psoriasis severity. Patients treated with pioglitazone alone achieved superior Psoriasis Area and Severity Index (PASI) 75 responses compared to placebo (OR 8.74; 95% CI, 3.76–20.31), and those on combination therapy (pioglitazone plus standard psoriasis treatment) also demonstrated enhanced outcomes (OR 4.64; 95% CI, 2.03–10.60) (Chang et al., 2020). Additional studies reported concurrent improvements in both metabolic parameters and psoriasis severity in overweight and obese patients treated with pioglitazone (Ghiasi et al., 2019; Lajevardi et al., 2015; Singh and Bhansali, 2016).

GLP-1 receptor agonists such as semaglutide and liraglutide, approved for obesity and diabetes, have also shown promise in treating skin disorders. A meta-analysis involving 32 psoriasis patients with type 2 diabetes across four trials reported that liraglutide therapy significantly decreased psoriasis severity (Chang et al., 2022).

A broader review of longitudinal cohorts and randomized controlled trials (RCTs) on GLP-1RAs revealed PASI improvement in four out of five studies (Ahern et al., 2013; Buysschaert et al., 2014; Faurschou et al., 2015; Lin et al., 2022; Xu et al., 2019). For instance, a RCT by Faurschou et al. (2015) in glucose-tolerant, overweight psoriasis patients did not show significant improvement in week 8. However, another RCT by Lin et al. (2022) involving 25 diabetic patients with a mean BMI of 24, demonstrated significant improvement in both PASI and Dermatology Life Quality Index (DLQI) at 12 weeks. Case reports on semaglutide also noted marked PASI improvement.

In HS, liraglutide treatment in a small cohort of 14 obese patients unresponsive to standard therapy resulted in clinical improvement, especially in those achieving significant weight loss (Nicolau et al., 2023).

## Limitations and Confounding Factors

Current evidence linking obesity, microbiome alterations, and inflammatory skin diseases is limited by several factors. Most studies are observational and cross-sectional, making causal inference difficult and leaving findings vulnerable to confounding by diet, medications, lifestyle, and genetic predisposition. Substantial methodological variability across microbiome studies—including differences in sampling sites, sequencing approaches, and analytic pipelines—further reduces reproducibility and complicates comparisons between cohorts. Obesity itself also acts as a major confounder, often obscuring disease-specific microbial signatures in HS and psoriasis. Additionally, geographic, ethnic, and anatomical variability limit generalizability. These constraints highlight the need for longitudinal, BMI-adjusted, and multi-omics studies to clarify mechanistic pathways and strengthen translational relevance.

## Conclusion

This review underscores the intricate interplay between obesity, gut and skin microbiota, and inflammatory skin diseases such as HS and psoriasis. Current evidence suggests that obesity is not only a metabolic and immunologic driver of skin inflammation but also a modifier of microbial communities, thereby amplifying disease onset, severity, and chronicity. These findings highlight the gut–skin–obesity interplay as a unifying framework linking systemic metabolic status with local cutaneous immune responses and microbial dysbiosis. These insights position the gut–skin–obesity axis as a unifying framework that links systemic metabolic status with cutaneous immune responses and microbial dysbiosis. Future research integrating longitudinal designs, BMI-adjusted analyses, and multi-omics approaches will be essential for identifying functional microbial pathways and advancing microbiota- and metabolism-targeted therapeutic interventions. Ultimately, a deeper understanding of these interconnected systems may enable more precise and effective strategies for managing obesity-associated inflammatory skin diseases.

## Figures and Tables

**Table 1.** Summary of studies investigating the impact of obesity on skin microbiota

Author	Sample size	Methods	Key findings	Limitation
**Healthy individuals**				
Brandwein et al. (2019)	822 skin samples	16S rRNA V4 sequencing	Skin microbiome beta diversity and relative abundance of *Corynebacterium* positively correlated with BMI.	BMI self-reported; cross-sectional design; no control for comorbidities; Specific skin area could not be identified
Rood et al. (2018)	31 obese and 27 normal-weight pregnant women	16S rRNA V1-V3 sequencing	In the mid-abdomen and Pfannenstiel area, obese individuals had higher levels of Firmicutes and Bacteroidetes, and lower levels of *Actinobacteria* compared to controls.	Small sample size; pregnant women only (reduced generalizability); potential confounders (pregnancy-related factors, surgical prep. antibiotics) not fully controlled
Vongsa et al. (2019)	20 women with high BMI (≥ 30), 20 with normal BMI	16S rRNA V1-V3 sequencing	Women with normal BMI showed *Lactobacillus*-dominant flora; those with high BMI had more *Finegoldia* and *Corynebacterium*, particularly in the vulvar region. No significant differences were noted in abdominal skin.	Limited to female subjects; region-specific sampling (vulvar/abdominal), potential confounders (ethnicity, diet, hygiene practices, and hormonal variation) were not fully adjusted.
Walker et al. (2020)	10 obese (BMI 35–50) postmenopausal women, 10 normal-weight (BMI 18.5–26.9) women	16S rRNA V1-V3 sequencing	Minimal differences in overall skin microbiome composition between groups (mid lower abdomen).	Very small cohort; postmenopausal women only (reduced generalizability); potential confounders (ethnicity, diet, hygiene products, sexual activity, antibiotic history and hormonal variation) not fully adjusted.
Ma et al. (2024)	198 healthy Chinese women	16S rRNA V3-V4 sequencing	Higher BMI associated with impaired skin barrier (increased TEWL, decreased pH), increased bacterial and fungal diversity. Overweight group had elevated Streptococcus, Corynebacterium, Malassezia, Candida abundance. Significant correlations observed between skin physiology and microbial composition.	Single anatomical site (face); cross-sectional design
**HS**				
Haskin et al. (2016)	632 HS patients	Bacterial culture of purulent drainage	The odds of detecting *Firmicutes* were 3.1 times higher in obese HS patients than in non-obese counterparts.	Culture-based (bias against unculturable bacteria); lack of control; potential confounders (antibiotic exposure and comorbidities) are not systematically controlled.

Abbreviation: BMI, body mass index; HS, hidradenitis suppurativa; RNA, Ribonucleic acid.

**Table 2.** Summary of longitudinal cohort studies and randomized controlled trials (RCTs) evaluating the effects of obesity-targeted pharmacologic interventions on psoriasis and hidradenitis suppurativa (HS)

Author	Study design	Sample size	Intervention	Key findings	Limitation
**Psoriasis: Liraglutide**					
Buysschaert et al. (2014)	Prospective cohort	7 patients with type 2 DM and psoriasis	18 weeks of exenatide (5 μg BID) or liraglutide (1.2 mg daily)	Mean PASI decreased from 12.0 to 9.2	Very small, uncontrolled case-series; Treatment & co-therapy heterogeneity; short follow-up
Ahern et al. (2013)	Prospective cohort	7 patients with type 2 DM and psoriasis	10 weeks of liraglutide (1.2 mg daily)	Median PASI decreased from 4.8 to 3.0; DLQI from 6.0 to 2.0	Small open-label study without controls; low baseline disease activity; confounding by metabolic changes and co-therapies
Faurschou et al. (2015)	RCT	20 psoriasis patients	8 weeks of liraglutide (1.2 mg daily) vs. placebo	No significant difference in PASI and DLQI between groups	Small sample size and short treatment duration; no significant PASI/DLQI benefit over placebo
Xu et al. (2019)	Prospective cohort	7 patients with type 2 DM and psoriasis	12 weeks of liraglutide (1.2 mg daily)	Mean PASI dropped from 15.7 to 2.2; DLQI from 21.6 to 4.1	Very small, uncontrolled study; short follow-up; potential confounders for metabolic and treatment regimens for diabetes
Lin et al. (2022)	RCT	25 psoriasis patients with type 2 DM	12 weeks of liraglutide vs. placebo	Significant PASI improvement in treatment vs. control group	Small, single-center, open-label trial; short follow-up; confounding by metabolic effects
**Psoriasis: Pioglitazone**					
Singh and Bhansali et al. (2016)	RCT	60 psoriasis patients with MS	12 weeks of pioglitazone vs. metformin vs. placebo	Significant improvement in PASI, PGA, and ESI with pioglitazone and metformin	Single center, open label study; short treatment window
Ghiasi et al. (2019)	RCT	60 psoriasis patients with MS	10 weeks of phototherapy + pioglitazone vs. phototherapy + placebo	Greater PASI reduction in pioglitazone group	Single center study; short treatment window; fixed-dose design
Lajevardi et al. (2015)	RCT	44 psoriasis patients	16 weeks of MTX + pioglitazone vs. MTX alone	PASI75 achieved in 63.6% (combo) vs. 9.1% (MTX alone)	Assessor-blinded only; single center trial with small sample size; male-predominant cohort
**HS: Liraglutide**					
Nicolau et al. (2023)	Prospective cohort	14 HS patients with obesity	12 weeks of liraglutide (3 mg)	Significant reductions in BMI, Hurley stage, and DLQI	Small sample size; short follow-up duration; lack of a control or placebo group

Abbreviation: DLQI, dermatology life quality index; DM, diabetes mellitus; ESI, erythema, scaling, and induration; MS, metabolic syndrome; MTX, methotrexate; PASI, psoriasis area and severity index; PGA, physician global assessment; RCT, randomized placebo-controlled trial.
